# Minimal Extrathyroid Extension (mETE) as the Only Risk Factor in Patients with Papillary Thyroid Carcinoma (PC): Its Clinical Impact on Recurrence and Outcome during Long-Term Follow-Up

**DOI:** 10.3390/biomedicines12020350

**Published:** 2024-02-02

**Authors:** Andrea Marongiu, Susanna Nuvoli, Andrea De Vito, Sonia Vargiu, Angela Spanu, Giuseppe Madeddu

**Affiliations:** 1 Unit of Nuclear Medicine, Department of Medicine, Surgery and Pharmacy, University of Sassari, 07100 Sassari, Italy; amarongiu2@uniss.it (A.M.); smfnuvoli@uniss.it (S.N.); sovargiu@tiscali.it (S.V.); giuseppe.madeddu@email.it (G.M.); 2 Unit of Infectious Diseases, Department of Medicine, Surgery and Pharmacy, University of Sassari, 07100 Sassari, Italy; andreadevitoaho@gmail.com

**Keywords:** papillary thyroid carcinoma, risk factors, minimal extrathyroid extension, follow-up, neck lymph node metastasis, distant metastasis, ^131^I SPECT/CT

## Abstract

Minimal extrathyroid extension (mETE) effect on papillary thyroid carcinoma (PC) prognosis is still debated even more so now that this factor has been removed in the 8th AJCC Edition, supporting the hypothesis that mETE is not associated with aggressive tumors. We retrospectively enrolled 91 PC patients (Group 1) submitted to total thyroidectomy and radioiodine ablation. At the time of the primary tumor surgery, mETE was ascertained in all patients with no other risk factors, such as multifocality, vascular invasion, neck and distant metastases, and aggressive histological variants. As controls, 205 consecutive matched PC patients (Group 2) without mETE and the aforementioned risk factors were enrolled. During the follow-up (average 8 years), 16/91 (17.58%) Group 1 patients and 15/205 (7.32%) Group 2 patients developed metastases (*p* = 0.0078). Cox regression analysis showed an increased risk of metastases in patients with mETE (HR: 2.58 (95% CI 1.28–5.22) *p* = 0.008). Disease-free survival (DFS) was significantly lower in patients with mETE than in controls (*p* = 0.0059). The present study seems to demonstrate that mETE can be associated with an aggressive PC and can be considered, even alone without other risk factors, an independent factor of unfavorable DFS. Thus, by excluding mETE in the 8th AJCC Edition, patient care and management could be compromised.

## 1. Introduction

The extrathyroid extension (ETE) in patients with differentiated thyroid carcinoma (DTC) has been defined as when tumor cells invade tissues and structures near the gland. In particular, extended ETE (eETE) represents the macroscopic tumor extension to subcutaneous soft tissue, trachea, larynx, esophagus, or recurrent laryngeal nerve, while in the minimal ETE (mETE) condition, extension to thyroid capsule, perithyroid soft tissue, and/or sternothyroid muscle invasion are microscopically detected at histology.

While the eETE is recognized as a predicted factor for tumor recurrence and mortality, the role of mETE as a risk factor for developing metastases and its impact on clinical outcomes during follow-up after thyroidectomy and radioiodine ablation is still a matter of debate [1,2,3,4,5,6,7,8,9,10,11,12]. Some authors retain that mETE has no impact on the disease behavior and prognosis, not representing a risk factor for developing metastases during the follow-up [11,13,14,15,16,17,18,19,20,21,22,23,24]. However, others do not agree with this interpretation and reported that mETE could play an important role in PTC patients, suggesting that this factor is associated with an increased risk for a poor outcome [10,25,26,27,28,29,30]. Moreover, mETE, in particular in patients with primary tumor ≥ 1 cm, can be considered a significant factor in the management of cervical lymph node (LN) metastasis in patients with PTC [31].

The risk stratification according to the ATA guidelines [32] considers the patients with mETE as patients with intermediate risk for developing metastases during the follow-up. In the face of this risk, the major guidelines for thyroid cancer suggest in these patients a more aggressive treatment such as total thyroidectomy, prophylactic dissection of LN of the central compartment, and subsequent radioiodine therapy.

However, recently, the 8th Edition of the American Joint Committee on Cancer (AJCC) [33] removed mETE from the definition of T3 disease when it is only detected on histological examination. This is in contrast to what has been reported in both the seventh and sixth editions, where mETE was categorized as T3, as well as in the fifth edition, where ETE, including both eETE and mETE, had been categorized as T4. Thus, the conflict remains whether the presence of mETE may still have significant importance in specifying TNM stage in patients operated for DTC and its impact on the management and outcome of the patients as a predictor of prognosis.

In light of controversial data reported in the literature, the present study aimed to further investigate the impact of mETE, ascertained microscopically at histology, on metastasis development and outcome during long-term follow-up. The study was carried out in a group of patients with papillary carcinoma (PC) with mETE, but without risk factors at the surgery of the primary tumor, such as eETE, vascular invasion, multifocality/multicentricity, neck and distant metastases, and aggressive histological PC variants. The data obtained in the patients with mETE have been compared with those observed in a control group of thyroidectomized PC patients without mETE and the same risk factors as above. 

## 2. Materials and Methods

### 2.1. Patients

Among a large PC patient group submitted to total thyroidectomy and radioiodine ablation therapy, 91 consecutive patients (Group 1) were retrospectively enrolled. In all of them, mETE had been ascertained at histological microscopic exam after surgery. The inclusion criteria were that the patients had no risk factors, such as eETE, vascular invasion, multifocality/multicentricity, neck LN metastases both in the central compartment and in the lateral cervical regions, distant metastases, and aggressive histological PC variants.

Table 1 illustrates the characteristics of 91 patients at the time of the primary tumor surgery: 69/91 patients were females, 22/91 were males, 48 were aged <55 years, and 43 were aged ≥55 years. The size of carcinomas was ≤10 mm (microcarcinomas-PTMC) in 20 patients (5 ≤ 5 mm and 15 > 5 mm) and >10 mm in 71 patients. According to the risk stratification system, based on the most recent American Thyroid Association (ATA) guidelines [32], these 91 patients with mETE were classified as intermediate risk. Based on the TNM system, considering the AJCC 8th [33], 20 patients were T1aN0M0, 33 T1bN0M0, 29 T2N0M0, and 9 T3aN0M0.

Two hundred and five PC consecutive patients (Group 2) shown in the same Table 1, were also retrospectively enrolled as controls, and these were matched for sex, age, and tumor size with Group 1 patients. All patients had undergone total thyroidectomy and radioiodine ablation in the same period as PC patients with mETE. In all cases, neither mETE nor the risk factors previously mentioned were ascertained in histology. Based on the TNM system, considering the AJCC 8th, 60 patients were T1aN0M0, 78 T1bN0M0, 51 T2N0M0, and 16 T3aN0M0.

In 64/91 patients of Group 1, surgery was performed because the presence of thyroid nodules was highly suspect for DTC at fine needle aspiration biopsy (FNAB) and was confirmed at histology (10 PTMC), while the remaining 27/91 patients were operated on for multinodular goiter and carcinomas identified only at the time of histology (10 PTMC).

In Group 2 patients, the cause of the surgery in 124/205 patients was the presence of predominant thyroid nodules suspect for cancer at FNAB, 19 of these PTMC. The reason for the surgery in the other 81/205 patients was the abnormal growth of multinodular goiter, and the carcinoma foci were identified only microscopically at histology and 41 of these were PTMC.

Thyroid antibodies (AbTg, AbTPO, and TRABs) were absent or under cut-off in the serum of the two patient groups, and histological abnormalities from thyroid autoimmune diseases in association with PC were not ascertained. Moreover, all patients were on euthyroid clinical status, thus excluding hyper or hypothyroidism current conditions, no patients being on specific pharmacological therapy before surgery.

The total thyroidectomies were performed according to the therapeutic strategy of choice in the University Surgery Department, and no patient had postoperative complications, such as recurrent laryngeal nerve invasion, hypocalcemia, etc. After surgery, the patients underwent radioiodine ablation.

Globally, removal of central neck LN was performed in 41 patients (19 in Group 1 and 22 in Group 2) and lateral LN in 9 patients (5 in Group 1 and 4 in Group 2) when these were suspected of cancer during imaging procedures before surgery or during operation, but metastases had not been revealed.

As shown in Table 1, the patients of the two PC groups with and without mETE are comparable and no statistical difference was ascertained.

Histology confirmed the diagnosis of suspected metastases during follow-up. In some instances, the absence of histopathological findings for suspected lesions was observed due to the difficulty in reaching the potential site of the lesions. When histology was unavailable, the diagnosis has been validated through close follow-up for about 120 months and an average of 96 months with clinical and imaging procedures as well as traditional periodic assessment of thyroglobulin serum levels. The lesions thus ascertained were considered recurrent metastases and not as persistent disease when they appeared at least 18 months after radioiodine ablation.

### 2.2. Methods

All patients were monitored in a long-term follow-up after thyroidectomy and subsequent radioiodine ablation. In particular, PC patients with mETE were followed-up for 96.42 ± 31.03 months and control patients without mETE for a mean period of 95.10 ± 33.39 months; the difference is not statistically significant (*p* = 0.749).

During follow-up, to evaluate metastasis status, the patients were monitored with common diagnostic procedures, such as clinical exams, neck ultrasound, ^131^I-Whole Body Scan (WBS), and single photon emission computerized tomography/computerized tomography (SPECT/CT). The latter two procedures were performed 24–48 h, and 72 h if necessary, after 185 MBq radioiodine diagnostic dose using a hybrid dual-head gamma camera in patients with hypothyroidism after L-thyroxine withdrawal or after recombinant thyroid stimulating hormone (rhTSH). Serum TSH levels were always over 50 µU/mL. Some patients, as complementary examinations, underwent CT or MRI and ^18^F-FDG PET/CT, and, when the lesions were accessible, FNABs by ultrasound were practiced. Sequential serum thyroglobulin and AbTg levels were assayed, at intervals of 6–9 months, by chemiluminescent immunoassay method; the detection limit of thyroglobulin assay is 0.1 ng/mL. The cut-off was considered <0.2 ng/mL during suppressive therapy and <1 ng/mL after TSH stimulation. The AbTg cut-off was 100 IU/mL.

By chemiluminescent immunoassay method, AbTPO (cut-off: 16 IU/mL) and TRABs (cut-off: 1.75 IU/L) were also assayed. The values of cut-off of all the antibodies do represent the upper limit of the normal levels, and the values are considered positive if above that limit.

All radioisotopic instrumental examinations were performed in the Nuclear Medicine Center of the University Hospital, the site of the present study. Four nuclear medicine physicians (A.M., S.N., A.S., and G.M.), were aware of the reason for the exams but unaware of the results of the other previous investigations. Interobserver variability was very low, and disagreements were resolved by consensus. The other imaging exams were performed in the Radiologic Center and the “in vitro” tests in the Central Endocrinological Laboratory of the same University Hospital.

### 2.3. Statistics

The normality of quantitative data was evaluated by the Shapiro–Wilk test. Quantitative variables were summarized with mean ± standard deviation (SD) or medians and 25–75° percentiles (IQR), whereas qualitative ones were by absolute and relative (percentages) frequencies. The Mann–Whitney test or Student *t*-test evaluated the quantitative variable subgroup differences. The differences for qualitative variables were evaluated by Pearson chi-square or Fisher exact tests. Categorical variables were assessed by the Fisher chi-squared test.

A Cox regression analysis was performed to test the association between the collected variables and the risk of metastasis. Metastases were considered a dependent variable. The significance was fixed as *p* < 0.05.

Kaplan–Meier curves were plotted to visually assess 10-year disease-free survival, using the log-rank test to assess the statistical difference between mETE and non-mETE patients. A two-tailed *p* < 0.05 was considered statistically significant. All statistical analyses were performed with STATA version 16.1 (StataCorp. LLC, College Station, TX, USA).

## 3. Results

The results of the long-term follow-up after surgery and radioiodine ablation therapy in both Group 1 and Group 2 PC patients who developed metastases are illustrated in Table 2.

In total, 16/91 (17.58%) patients of Group 1 developed metastases; 10/16 patients were females and 6/16 males, 9/16 were aged <55 years, and 7/16 ≥ 55 years; 4/16 patients had a tumor size ≤ 10 mm (PTMC), all of these with a size > 5 mm, and 12/16 with a size > 10 mm.

As illustrated in Table 2, as regards TNM, the comparison of the percentages of patients with metastases compared exclusively to the total number of patients (Group 1 and Group 2) who developed metastases with the same TNM category did not show statistical significance (*p* = 0.747). Even when the percentages of patients with metastases mentioned above were correlated to the total number of patients with the same TNM (not reported in the table), the comparison excluded a statistically significant difference (*p* = 0.829).

Thirty-three metastases were ascertained in the 16 patients of Group 1 during the follow-up. In 15/16 cases, 24 LN metastases were in the neck, 9 of which were laterocervical (LTC), 12 paratracheal (PT), and three submandibular (SM), including the four patients with PTMC, while in the remaining 1/16 patients, only one bone metastasis (cervical spine) was found. Two of the 15/16 patients, besides neck LN metastases, also had distant metastases. In particular, in one of these with LTC metastasis, one LN metastatic lesion in the superior mediastinum was ascertained; in the other patient, besides seven neck LN metastases, three lung and three bone metastatic lesions (one skull, one homerus and one femur) were found. This patient was the only one of 16 patients who had thyroid cancer-specific death, despite a second surgery on the neck and two further radioiodine ablations for both local and distant metastases during follow-up.

In 205 Group 2 patients, metastases occurred in 15/201 (7.32%) cases, ten females and eight males, eight aged <55 years and seven ≥ 55 years. Ten patients had tumor size >10 mm, while 5 ≤ 10 mm, two of the latter ≤ 5 mm.

Globally, 23 metastases were identified in 15 patients: two for each patient were found in the neck in four cases, one for each patient in the neck in nine cases, and one lesion in the neck together with one in the lung and one in the bone (sternum) in another case. In the remaining patient, one lesion in the neck, one in the superior mediastinum and one in the bone (spine) were ascertained.

The patients of Group 1 with mETE had a higher age and tumor size with respect to the patients of Group 2 without mETE, but the difference was not statistically significant.

The difference in the percentage of patients who developed metastases between Group 1 and Group 2 was statistically significant (*p* = 0.0078).

Except for the aforementioned patient who died of thyroid cancer, all the other patients of both groups are still alive and remain under observation.

As shown in Table 3, comparing the different variables of these patients, only mETE, at Cox regression analysis showed an increased risk of metastasis appearance during follow-up: HR: 2.58 (95% CI 1.28–5.22), *p* = 0.008.

Furthermore, in the mETE patients, metastasis appearance occurred in a shorter time in comparison with the control patients without mETE; however, the difference between median values of the two groups was not statistically significant (24 (IQR 22.5–35) months vs. 28 (IQR 24–34) months, *p* = 0.378).

**Figure 1:**
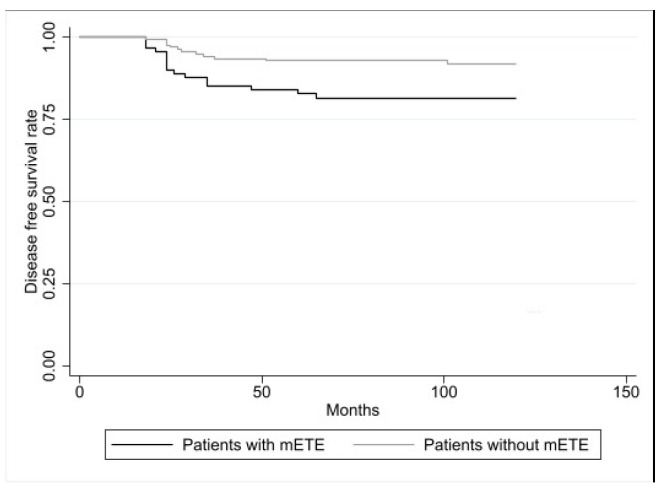
Disease-free survival (DFS) in PC patients with mETE (*n*. 91) and in PC patients without mETE (*n*. 205). According to the log-rank test, the difference was statistically significant (*p* = 0.0059).

Evaluating the disease-free survival (DFS) after ten years, this was significantly lower in the patients with mETE with respect to the patients without mETE (82% vs. 92%, *p* = 0.0059), as reported in Figure 1.

**Figure 2:**
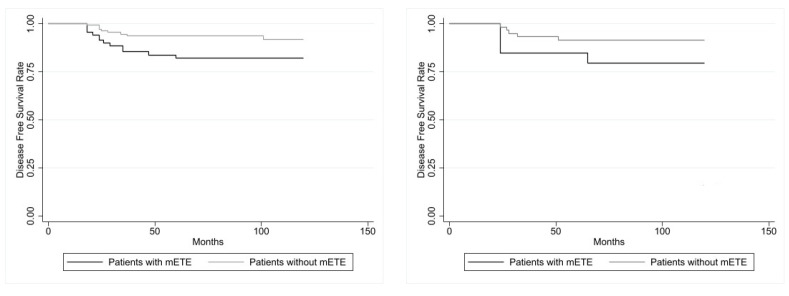
At (**left**): disease-free survival (DFS) in the 71 PC patients with mETE and in the 145 PC control patients with carcinoma >10 mm showed a statistical difference (*p* = 0.0181, calculated with log-rank test). At (**right**): disease-free survival (DFS) in the 20 PC patients with mETE and in the 60 PC control patients with PTMC (≤10 mm) did not show statistical difference (*p* = 0.1526, calculated with log-rank test).

As illustrated in Figure 2, evaluating DFS of both Group 1 and Group 2 patients with tumor size > 10 mm, this parameter was significantly reduced (*p* = 0.0181) according to the log-rank test, while in the patients with PTMC, this parameter showed no statistically significant difference (*p* = 0.1526).

Subdividing the patients with PTMC according to the different sizes ≤ 5 and >5 mm, DFS was significantly (*p* = 0.034) reduced in the patients with size > 5 mm, but it was not significantly (*p* = 0.370) reduced in those with size ≤ 5 mm.

## 4. Discussion

The studies reported in the literature on the role of mETE during the follow-up of patients thyroidectomized for papillary thyroid carcinoma underline that this aspect is still strongly debated. In particular, microscopic extrathyroid extension in peri-thyroidal soft tissue is not explicitly mentioned in the AJCC 8th Edition [33] notwithstanding this infiltration is relatively frequent, since some authors retain that it does not represent a relevant prognostic factor [34], thus not justifying its inclusion in a separate category. This viewpoint had been confirmed by other authors before the 8th Edition of the TNM classification was published [7,11,18,34] but not accepted by others [31,35].

After the publication of the AJCC 8th Edition, many authors reported the results of their studies that still underlined the controversies on this problem.

According to a study conducted to comment on the stringent criteria proposed by the 8th Edition of the AJCC Staging System Manual, these were considered appropriate, supporting what was reported in the AJCC. These stringent criteria could decrease variability between observers and increase consistency in the diagnosis and staging of thyroid carcinoma [36].

In further support of what is reported in AJCC 8th Edition on the absence of mETE in the manual, some authors reported that the tumor size might be considered a more important factor than the presence of mETE in disease recurrence. Additionally, mETE was not a significant predictor of DFS, locoregional recurrences, and distant metastasis detection on multivariate analysis [22]. Another study showed that patients with mETE and those without mETE had the same recurrence rate or metastasis development, suggesting that intensive treatment may not be necessary for patients with mETE [23]. In particular, the study showed that age > 55 years did not have a significant impact on the risk of disease. In other studies, it has been observed that radioiodine therapy had a similar response rate in PTC patients regardless of the presence of mETE, although there was a high frequency of LN metastases [37]. According to the results obtained by other authors who reported that mETE increases the risk of recurrence in DTC patients, it has been observed that the absolute increase is small and, in addition, mETE has no impact on disease-related mortality [38]. Although in some studies mETE with an invasion of strap muscles did not seem to be a marker for poor prognosis, in other series of patients with age ≥ 55 years and large tumor size (>2 cm), the overall survival was impaired [39]. It has also been reported that mETE is a poor prognostic factor in tumors larger than 15 mm, but not in those smaller, which, without other unfavorable characteristics, should be classified as low risk [40].

However, based on the results obtained in other studies, mETE seems to represent an independent intermediate risk factor for recurrence except when it is identified in PTMC without LN metastases [12]. Other authors have reported that mETE may be associated with an increased risk of poor cancer-specific survival and overall survival, suggesting that it should be further investigated whether removing mETE from the last AJCC Staging System Manual is correct [25]. Furthermore, in other research, mETE was significantly linked to poor outcomes and the authors concluded that the TNM Staging System may need to be modified in the future due to this result [41]. It has been reported that even a small increase in thyroidal extension can lead to a significant increase in the risk of compromised survival. Therefore, mETE seems to be a reliable indicator of relapse-free survival and should be incorporated into the therapeutic thyroid cancer protocol [28] and thus included in a new AJCC Staging System Manual.

Considering the currently hot topic of debate, in the present retrospective study, the relationship between mETE and the prognosis of PC has been further evaluated during long-term follow-up of the affected patients after thyroidectomy and radioiodine ablation. A group of PC patients with no mETE was also included as controls, all of whom had been submitted to total thyroidectomy during the same period as patients with mETE.

Both groups of patients were exempted from the risk factors previously mentioned at the time of the primary tumor surgery, and age, gender, tumor size, histology, anatomic structure, and TNM did not differ significantly between patients with or without mETE.

During follow-up, it has been found that PC patients with mETE had a significantly higher rate of disease progression than PC matched control patients without mETE, even though their tumor characteristics were similar.

Compared to control patients, those with mETE had a significantly higher percentage of LN metastases. In addition, three patients with mETE and two belonging to the control group had distant metastases.

Moreover, patients with mETE had a significantly shorter DFS after ten years than those who did not have mETE. Additionally, patients with mETE experienced a faster occurrence of metastases than those without mETE. However, the difference in median values between both groups was not statistically significant.

The identification of metastases during follow-up is typically achieved through the use of traditional and highly sophisticated diagnostic imaging procedures, and in all patients, ^131^I SPECT/CT has been included with high performance, confirming the reliable results of this procedure reported in the literature [42,43,44,45] also in patients with PC associated to thyroid autoimmune diseases [46,47].

Furthermore, in PC patients with mETE who had metastases, it may not be excluded that mETE alone could be a predictive risk factor for the development of metastases, potentially leading to worse disease prognosis and undesirable outcomes. Confirmation of these data was achieved through a Cox regression analysis that compared patients with and without mETE.

PC with mETE was found to be more aggressive than PC without mETE in the series of patients in the present study. Despite this, during the study period, only one patient of mETE died from a thyroid tumor, but otherwise, there was no relationship between cancer mortality and mETE.

Other authors also observed the same results obtained in this study regarding the aggressiveness of PC in patients with mETE [25,28,41]. However, the information obtained was in opposition to what was observed by others in several studies, which indicated an excellent prognosis and favorable disease-free survival for the patients [22,23,37,38,39,40] regardless of the presence of mETE. The exact reason for these contradicting results remains unclear.

The majority of PC patients with mETE who developed metastases were women, aged <55 years, and had a carcinoma size that was above 10 mm. The patients were also represented by PTMC in 25% of patients, and all of these were larger than 5 mm.

Regarding PTMC, it is widely known that this type of tumor is a disease with minimal invasiveness and a favorable long-term prognosis; however, PTMC has the potential to be more aggressive in certain patients, particularly when it involves multifocal and bilateral involvement, metastases in the neck LN, and distant metastases [48]. Thus, PTMC identification is necessary for the earliest treatment, and long follow-up surveillance is appropriate given that the recurrence rate lasts for many years, as validated in previous studies on this type of tumor [48].

Like classic PC with size > 10 mm, the role of mETE in PTMC as a risk factor is unclear and it is still debated with controversial results. Some authors [49] have reported that mETE did not show any impact on recurrences in patients with PTMC and there was no difference in DFS between patients with or without mETE (*p* = 0.671). No significant effect on recurrence rate in patients with PTMC has been also confirmed in a meta-analysis [38]. Moreover, patients with less than 5 mm have been reported to be associated with a low recurrence risk, leading to a better prognosis, which may allow clinicians to select less aggressive management strategies [50].

However, other authors [51] consider mETE as a factor in worsening prognosis with the presence of metastatic LN and a lower rate of DFS (*p* = 0.034). Moreover, mETE was reported as an independent risk factor for cancer recurrence for both neck LN and distant metastases in PTMC [52]. Still, other authors observed that mETE was independently related to LN metastases compared to patients without mETE, thus suggesting that this factor should be considered in the management of patients with single PTMC [53]. In the present study, mETE in patients with carcinomas smaller than 10 mm appeared not to influence DFS, unlike carcinomas over 10 mm. However, when the sizes were considered in the patients with PTMC, it was observed that those larger than 5 mm seemed to significantly affect the patient outcomes when compared to the carcinomas smaller than 5 mm. However, these data must be considered with caution due to the small number of patients.

Despite the absence of several risk factors at the time of the primary tumor surgery, as mentioned earlier, there were 17.58% of patients with mETE who underwent metastases; this percentage was significantly (*p* = 0.0078) higher than that of patients without mETE (7.32%).

Understanding tumor aggressiveness in certain instances, like those observed in this study, may require more research into a large number of PC patients with mETE. This latter factor alone appears to be the responsible one in these patients where other risk factors are absent, but the mechanism remains unclear.

Some limitations must be evidenced in the study:

The study is retrospective and only involves one center, which is the first thing to note. In addition, some information can be lost, and the study involves a modest number of patients because the exclusion criteria were very narrow, with only patients with mETE excluding the other aggressive risk factors.

Second, even though the average follow-up period is long, it may not be sufficient to detect occult metastasis if these only emerge during clinical and imaging examinations because metastases may occur later.

Third, the absence of histological findings in certain metastases is a limitation due to either the challenge of finding their sites or the unavailability of invasive intervention because of ethical reasons. The nature of the metastases can only be validated through long-term follow-up that includes clinical and imaging procedures performed sequentially, along with thyroglobulin changes, in these patients.

## 5. Conclusions

The results of the present study suggest that mETE may have an unfavorable effect on the outcome of the patients during the follow-up after thyroidectomy and radioiodine ablation, with a significantly different disease progression in patients with mETE than PC cases without mETE, and with a significantly lower DFS. These data, which have been confirmed by some authors, but not by others, suggest that even mETE alone may be a significant predictor factor for an increase in the risk of developing metastases in the future and hold greater significance since other risk factors were not found during the surgery of the primary tumor.

However, given the multiple contradictory findings on these issues, it is necessary to conduct more research on a larger number of patients, perhaps in prospective studies, to elucidate the various pathological mechanisms involved in the relationship between mETE and the prognosis of PC.

In closing, the present investigation demonstrates that mETE by itself is linked to an aggressive PC and is an independent factor of unfavorable DFS. Thus, excluding mETE in the 8th AJCC Edition could compromise patient care and management. Therefore, our data support the inclusion of mETE in the risk stratification model, such as in the previous 7th AJCC Edition, and, in addition, suggest carefully observing and following up in PC patients with mETE. In particular, a close observation of the patients with PTMC would also be necessary, especially those with a size over 5 mm, which was identified in a significant number of patients who developed metastases in the present casuistry.

## Figures and Tables

**Table 1:** Demographic and histological characteristics of 91 patients with papillary carcinoma (PC) with minimal extrathyroid extension (mETE) and 205 patients with PC without mETE who served as controls, at the time of the primary tumor surgery.

	PC Patients with mETE	PC Patients without mETE as Controls	*p*-Value
	(91 Patients)	(205 Patients)	
**Age (years)**	48 (52.75%) < 55	124 (60.49%) < 55	0.213
	43 (47.25%) ≥ 55	81 (39.51%) ≥ 55	
**Age (years), mean ± SD**	52.79 ± 14.09	49.90 ± 14.64	0.110
**Sex (F/M)**	69 (75.82%)/22 (24.18%)	159 (77.56%)/46 (22.44%)	0.743
**Histology**	Classic variant 82 (90.1%) Follicular variant 9 (9.9%)	Classic variant 175 (85.37%) Follicular variant 30 (14.63%)	0.265
**Tumor size (mm)**	20 (21.98%) ≤ 10 71 (78.02%) > 10	60 (29.27%) ≤ 10 145 (70.73%) > 10	0.193
**Tumor size (mm), mean ± SD**	20.08 ± 11.87	17.49 ± 12.42	0.090
**Thyroid nodule (*n*.)**	64 (70.33%)	124 (60.49%)	0.105
**Multinodular goiter (*n*.)**	27 (29.67%)	81 (39.51%)	
**TNM (AJCC 8th)**			0.431
T1a N0 M0	20 (22.0%)	60 (29.3%)	
T1b N0 M0	33 (36.2%)	78 (38.0%)	
T2 N0 M0	29 (31.9%)	51 (24.9%)	
T3a N0 M0	9 (9.9%)	16 (7.8%)	

**Table 2:** Demographic and histological characteristics of 16/91 patients with mETE of Group 1 and 15/205 patients without mETE of Group 2 who developed metastases during long-term follow-up after surgery and radioiodine ablation therapy. All patients with mETE were classified as intermediate risk according to ATA classification and TNM according to AJCC 8th at the surgery of the primary tumor.

	16 PC Patients with mETE (Group 1)	15 PC Patients without mETE (Group 2)	*p*-Value
**Age (years)**	(9 < 55/7 ≥ 55)	(8 < 55/7 ≥ 55)	0.870
**Age (years) mean** **± SD**	54.50 ± 13.35	50.67 ± 15.68	0.469
**Sex (M/F)**	6/10	5/10	0.809
**Tumor size (mm)**	(4 ≤ 10/12 > 10)	(5 ≤ 10/10 > 10)	0.704
**Tumor size (mm) mean** **± SD**	20.56 ± 13.00	14.73 ± 8.98	0.159
**Histology**	16 classic variant	15 classic variant	
**TNM (AJCC 8th)**			
T1a N0M0 T1b N0M0 T2 N0M0 T3a N0M0	4 (25.0%) 6 (37.5%) 4 (25.0%) 2 (12.5%)	5 (33.3%) 7 (46.7%) 3 (20.0%) 0	0.747
**Metastatic lesions (*n*.)**	33	23	
**Neck LN metastases (*n*.)** LTC PT SM SC Mediastinal LN metastases	9 12 3 0 2	6 6 4 3 1	
**Distant metastases**			
Lung Bone	3 4 (1 spine, 1 skull, 1 homerus, 1 femur)	1 2 (1 spine, 1 sternum)	

LN: lymph node; LTC: laterocervical LN metastasis; PT: paratracheal LN metastasis; SM: submandibular LN metastasis; SC: supraclavicular LN metastasis.

**Table 3:** Cox regression analysis to assess the relationship between demographic and histological characteristics and 120 months of metastasis development.

	HR (95% CI)	*p*-Value
**Age (years)**	1.01 (0.98–1.03)	0.473
**Age < 55**	0.87 (0.43–1.77)	0.711
**Female gender**	0.52 (0.25–1.09)	0.086
**Tumor size**	1.00 (0.97–1.03)	0.821
**Carcinoma ≤ 10 mm**	1.06 (0.49–2.30)	0.882
**mETE**	2.58 (1.28–5.22)	0.008

## Data Availability

The data that have been presented in this study are available on reasonable request from the corresponding author.
